# A Case of Delayed Diagnosis of Moyamoya Disease After Recurrent Strokes

**DOI:** 10.7759/cureus.6446

**Published:** 2019-12-22

**Authors:** Oranus Mohammadi, David Krieger, Ifrah Butt, Mauricio Danckers

**Affiliations:** 1 Internal Medicine, Aventura Hospital and Medical Center, Aventura, USA; 2 Neurosurgery, Aventura Hospital and Medical Center, Aventura, USA; 3 Critical Care Medicine, Aventura Hospital and Medical Center, Aventura, USA

**Keywords:** moyamoya disease, recurrent stroke, encephaloduroarteriosynangiosis

## Abstract

We present a case of a 58-year-old man with delayed diagnosed moyamoya disease who underwent encephaloduroarteriosynangiosis (EDAS) procedure.

This patient with a history of three strokes presented to our facility with new left facial droop. Neurological examination revealed left facial droop and hemiparesis. Brain magnetic resonance imaging (MRI) described right frontal lobe acute ischemia. Head computed tomography (CT) angiography revealed bilateral supraclinoid internal carotid artery (ICA) occlusions. Cerebral angiography demonstrated diffuse intracranial vascular irregularity with stenosis, more above the bilateral supraclinoid ICAs and the right middle cerebral artery (MCA) suggestive of moyamoya disease. Due to the lack of MCA patency, he underwent EDAS. Superficial temporal artery (STA) was dissected inferiorly and the posterior branch was bipolared, then STA was movable. A bur hole made at the superior and inferior portion along the STA. Dura was opened, and STA was brought on top of the pia. His facial droop gradually improved after that. Nine months later, no new strokes reported.

Moyamoya disease is a rare neurovascular disorder characterized by narrowing and occlusion of the ICA branches. Its symptoms include recurrent ischemic/hemorrhagic strokes. Incidence in Hispanics has not been studied. The gap between the first manifestations and disease progression is one to eight years. Its diagnosis is often delayed. Our patient had recurrent strokes for five years. Despite therapy with antiplatelets, new ischemic stroke brought him to our institution. Rate of recurrent strokes despite antiplatelets was reported 10.3% per year. Brain CTs and MRIs had failed to detect strokes’ etiology. Catheter-directed angiography is the gold standard test for diagnosis of moyamoya disease. Antiplatelet alone is ineffective and surgery is the effective method to prevent further strokes, although there are no studies in adults regarding the efficacy of indirect revascularization. In direct revascularization, usually STA anastomoses to MCA. Indirect method works through the development of leptomeningeal collaterals. Postoperative complications are infarction and hyperperfusion syndrome. Seong-eun et al. proposed that modified EDAS is simpler with less complications in comparison with direct revascularization. Some other studies showed higher chance of stroke in indirect method versus direct technique.

In conclusion, it is important to consider moyamoya disease as a differential diagnosis in patients with recurrent strokes.

## Introduction

Moyamoya disease is a rare progressive condition with narrowing and occlusion of internal carotid artery (ICA) terminal ends [1]. A total of 1,063 moyamoya cases have occurred worldwide excluding Japan. The prevalence of the disease in Japan is 6.03 per 100,000 individuals, although the incidence of 0.086/100,000 is reported in the United States [2]. Typical presentations are stroke or transient ischemic disease. Encephaloduroarteriosynangiosis (EDAS) is a surgical intervention technique that helps to improve the vascular flow [3]. We present a case of a 58-year-old man with delayed diagnosed moyamoya disease who underwent successful EDAS procedure with good neurological outcomes. 

## Case presentation

**Figure 1:**
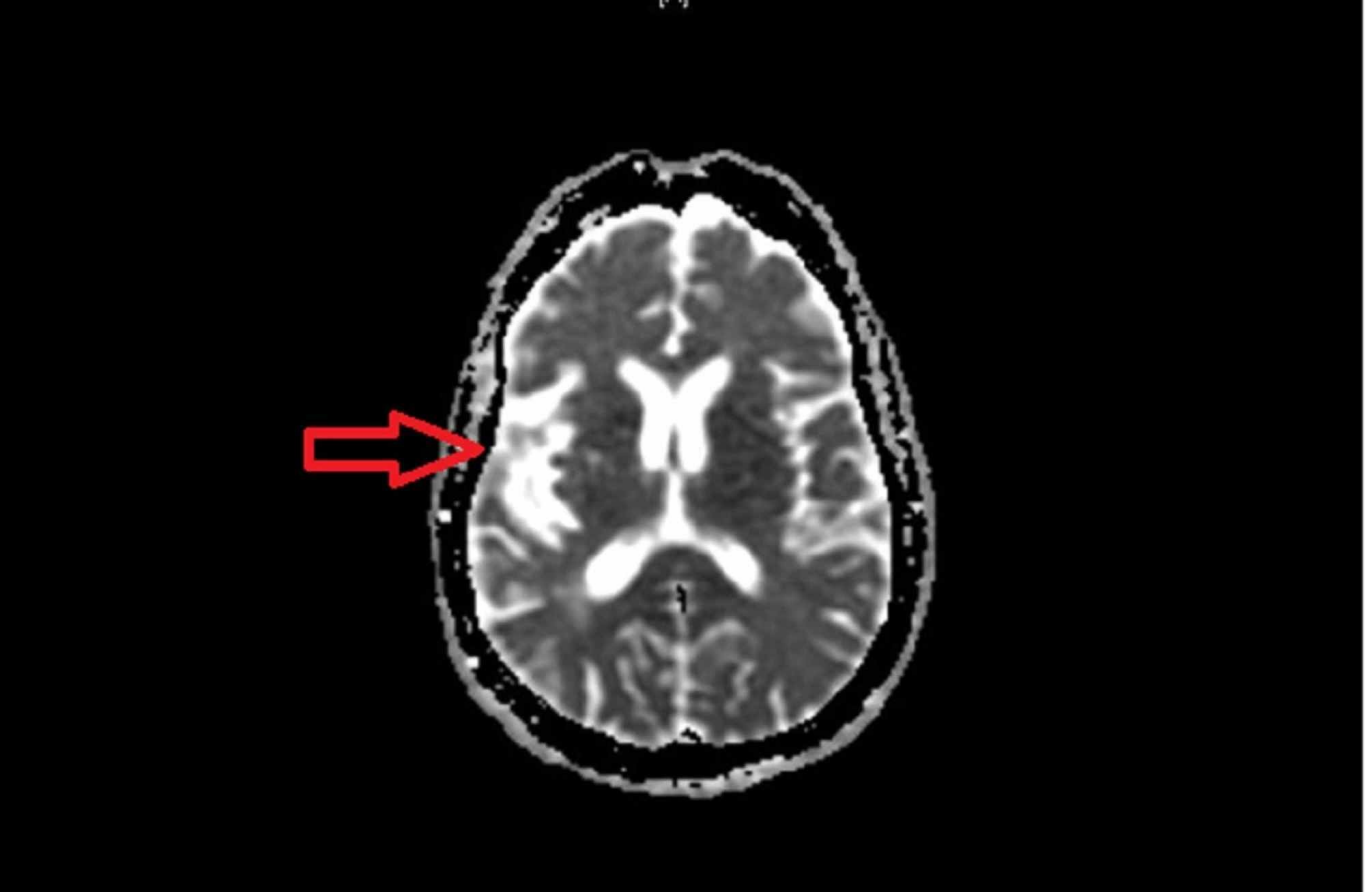
Brain MRI showed acute ischemia in the mid and post right frontal lobe

A 58-year-old Hispanic man with a history of coronary artery disease and three episodes of strokes with residual left upper and lower extremity weakness presented to our facility with a new onset left-sided facial droop. He reported facial numbness, dysarthria, and blurred vision. His neurological examination revealed left-sided facial droop, positive pronator drift, and left-sided hemiparesis, as well as positive left Babinski sign. Brain magnetic resonance imaging (MRI) without contrast described a patchy region of acute ischemia in the mid and posterior right frontal lobe, predominantly peripherally and cortically based, measuring 3.8 cm x 3 cm (Figure 1).

**Figure 2:**
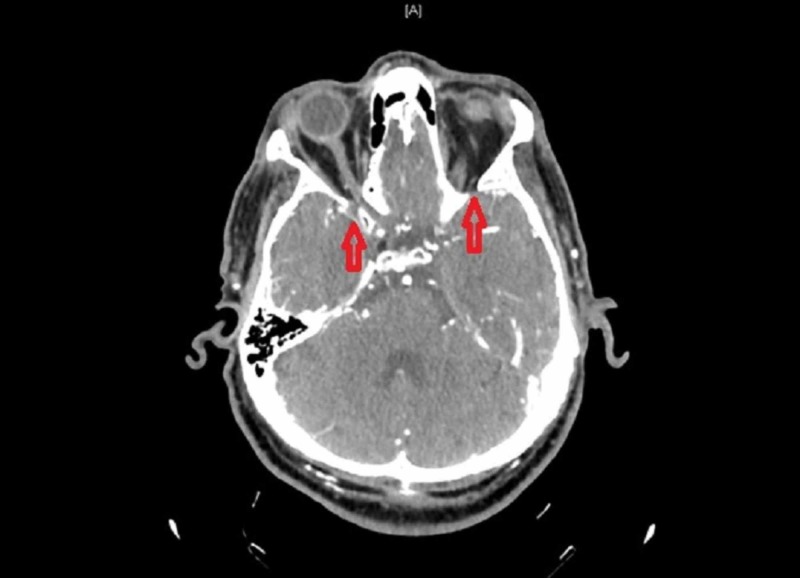
Neck and head CT angiography showed bilateral ICA occlusion ICA, internal carotid artery

Neck and head computed tomography (CT) angiography revealed bilateral supraclinoid ICA occlusions (Figure 2).

**Figure 3:**
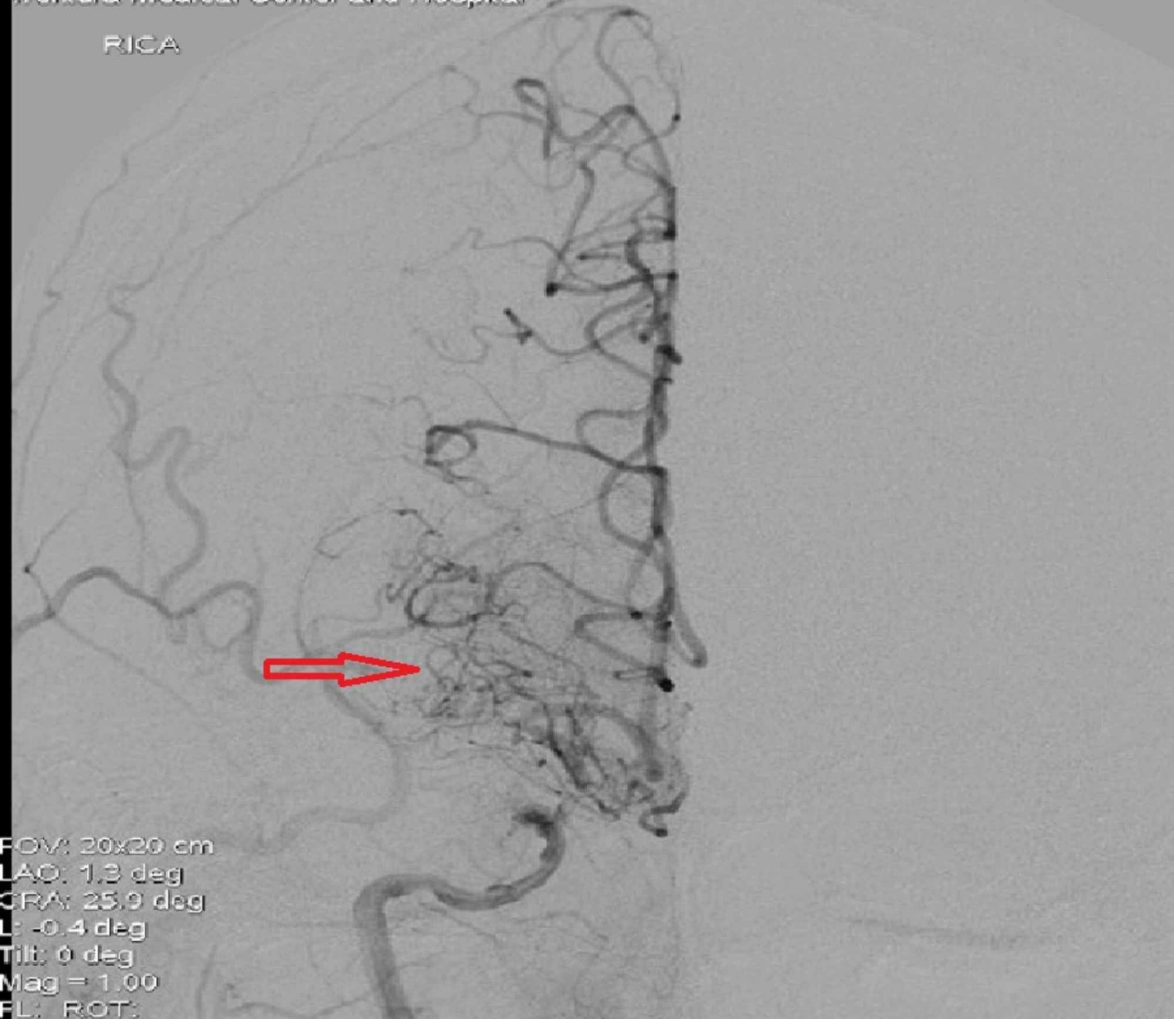
Cerebral angiography showed diffused intracranial vascular irregularity with stenosis

Cerebral angiography demonstrated diffuse intracranial vascular irregularity with stenosis, more pronounced above the bilateral supraclinoid ICAs and on the right middle cerebral artery (MCA) (Figure 3).

Imaging findings were suggestive of moyamoya phenomenon. Superficial temporal artery (STA)-MCA vascular bypass was not an option due to the lack of right MCA patency. Therefore, he underwent right-sided EDAS procedure. STA was dissected inferiorly to see it at just above the root of the zygoma. Superiorly, three branches were identified: one frontally, one temporally, and one superiorly. Even after full dissection, the STA was still not completely movable. The smallest posterior branch was bipolared and cut, and therefore there was more mobility in the STA. The other branches were all dopplerable. The STA was then again dissected completely free and moved anteriorly. Subperiosteal dissection was done. A perforator drill was used to makde a bur hole at the superior portion of the incision, and then one at the inferior portion of incision along the course of the STA. The burr holes were connected with a footplate, and the bone was removed in one piece. The dura was tented up and opened. The STA was brought on top of the brain. There was still some tension on the STA. Therefore, STA was further dissected at the level of the root of zygoma and at the superior branches. There was still tension on the STA, and therefore again it was dopplered inferiorly and superiorly. The frontal branch of the STA was bipolared and cut. At this point, the STA was completely free superiorly and inferiorly with good Doppler signals superiorly and inferiorly. The STA was brought in and laid down on top of the brain. The STA was on top of the pia without tension. No complications noted intraoperatively. Postoperatively, his facial droop gradually improved, but the residual left-sided weakness remained unchanged. He was discharged home with levetiracetam and aspirin. Follow-up of nine months later revealed no evidence of new stroke recurrence. 

## Discussion

Moyamoya disease is a progressive, chronic neurovascular disorder characterized by the narrowing and occlusion of the ICA terminal branches [4,5]. Clinical manifestation includes recurrent ischemic or hemorrhagic strokes. It affects mostly the Japanese population [6]. The incidence in the Hispanic population has not been studied. The incidence of this disease is bimodal, first at 10-20 years and second at 35-50 years [7]. The average gap between the age of first clinical presentation and disease progression is 1.5 to 8 years [8]. Its diagnosis is often delayed. Our patient had a history of recurrent ischemic strokes for more than five years. Despite secondary prevention therapy with antiplatelets, new ischemic stroke brought him to our institution. One study revealed the rate of recurrent strokes in patients on antiplatelet therapy was 10.3% per year [9]. Brain CT and MRI brain had failed to detect the etiology of his recurrent ischemic stroke. Catheter-directed angiography is the gold standard test for diagnosis of moyamoya disease [10]. Antiplatelet therapy alone is ineffective in the treatment of patients with moyamoya disease, and surgical intervention is the only method to prevent further neurological damage. 

Surgical revascularization has shown to be an effective treatment against cerebral ischemia by producing regional blood flow augmentation, but there are no controlled studies in adult population regarding the efficacy of indirect revascularization. Surgical interventions are three types: direct, indirect or combined. In direct revascularization technique, usually STA anastomoses to MCA [11]. Indirect bypass surgery works through the development of leptomeningeal collaterals [12]. Of all surgical cases, 35.1% were indirect revascularization including EDAS. Potential postoperative complications are cerebral infarction and hyperperfusion syndrome [10]. Seong-eun et al. proposed that modified EDAS is a simpler technique with less complications in comparison with direct revascularization [13]. Some other studies showed higher chance of stroke in indirect method in comparison with the direct technique [14]. The risk of perioperative strokes is 2.6% [15]. 

## Conclusions

It is important to consider moyamoya disease as a differential diagnosis in patients with recurrent strokes. The diagnosis is usually delayed, especially if patients present in older age. Brain CT and MRI cannot detect this medical condition. Catheter-directed angiography is the best tool if moyamoya disease is suspected. Combined antiplatelet therapy with revascularization can prevent further strokes.
